# A manually interpreted geo-dataset of building footprints in complex urban poverty areas across the globe

**DOI:** 10.1038/s41597-026-07945-2

**Published:** 2026-08-03

**Authors:** Nicolas J. Kraff, Michael Wurm, John Friesen, Henri Debray, Hannes Taubenböck

**Affiliations:** 1 German Aerospace Center, Earth Observation Center, 82234 Oberpfaffenhofen, Germany; 2 Institute for Geography and Geology, Julius-Maximilians-Universität Würzburg, 97074 Würzburg, Germany

## Abstract

Automated delineation of settlements at the level of single buildings is advancing on global scale due to developments in image processing techniques. However, in morphologically complex poverty areas (e.g., slums or informal settlements) automated image classification reaches limits. This gap of systematic, accurate geodata hinders the impetus of ‘better data for better decisions’ by the UN Sustainable Development Goals. To bridge this gap, we present a dataset of >320,000 building footprints derived from satellite imagery in 44 poverty areas across the globe. We use ‘Manual Visual Image Interpretation’ for consistent delineations of rooftops proxying building footprints. The method has been applied to 1. *classify* different *morphological types* representing poor living environments based on spatial features; 2. *document multitemporal* dynamics at different spatial scales; and, 3. *assess interpreter-related uncertainties* across interpreters. We release these data along with interpretation guidelines and validation. We provide a robust empirical basis for the global systematization and comparative analysis of settlement forms proxying poverty, while offering training and validation data for automated image classification.

## Background

The United Nations Sustainable Development Goal 11.1.1 calls for combating inhuman living conditions in slums and informal settlements, which are home to more than 1.1 billion people globally^1^. A better understanding aiming at a global repository of these areas^2,3^ is essential to address their precarious circumstances. However, official cadastral information, census statistics and formalized spatial planning are usually absent for these areas^4,5^, reflecting their informal character. Thus, it is essential to assess these areas’ urban built-up environment, respectively *morphology*. The term is defined as the study of the spatial form of cities through the configuration of elements (e.g., “taxonomic units”^6,7^ or “ontological”^8^) such as plots, streets, and especially buildings and building block, while also capturing the function of infrastructure that organizes how populations and buildings are concentrated in urban space (e.g.^9,10^).

Despite initial qualitative and quantitative approaches to globally classify poverty areas^2,4,8,11–14^, openly available, sufficiently accurate and consistent data at large scale for poverty-related settlements remain scarce. To address this gap, various research fields focus on the physical built environment, where morphology of the built-up environment serves as a proxy for deprivation.

To avoid terminological limitations^15^, these morphologies are often subsumed under umbrella terms such as “deprived areas”^16^ or “Arrival Cities”^4,17^. In this study, we use the term *poverty areas*.

Poverty areas are characterized by multifaceted morphologies, including high building densities, very often heterogeneous structures, and irregular alignments^2,18–20^. Their variability is extensive across continents, countries, and even within single cities^4,21^ and over time^22–24^. No universal repository and comprehensive characterization exist, and interdisciplinary data are strongly demanded^25,26^. Remote sensing provides an essential perspective to capture these environments systematically. Yet, the high morphological complexity of these built environments challenges automated processes, which often fail to achieve high accuracies at the single-building level^27–29^. The lack of reliable training/reference data in turn hampers the development of improved classification approaches, reinforcing a vicious circle.

Manual Visual Image Interpretation (MVII) remains a crucial method for generating reliable reference data in these environments, for example as training labels for machine learning^5,30–32^. These areas’ complexity arises from spectral mixing, dense alignments, shadow effects, and the noisy textures created by tightly arranged buildings^5,33–38^. This is especially challenging for remote-sensing based automated delineation approaches. Despite advances in automation, human interpreters still achieve the highest accuracies in identification and delineation of buildings^5,33,34,37^ in these areas. The dataset published here is derived from data generated in the following publications**:** firstly, we applied MVII to delineate informal from formal areas^18,39^, empirically showing the morphological differences in Mumbai, India; secondly, we developed a global categorization of a wide spectrum of settlement morphologies proxying the urban poor^4^; thirdly, we documented temporal settlement changes, highlighting persistence and transformation^22^. We finally quantified interpreter-related uncertainties, demonstrating both, the subjectivity and reproducibility of the method^40^.

This geo-dataset^41^ on the spatial scale of single-buildings is based on nine years of work [2011–2020]^4,18,22,40^ and we provide it free of charge for the first time to the scientific community. In contrast, global datasets either provide data on aggregated level (e.g.^13^ by the aggregation to 100 × 100 meter grids), exclude poverty areas at the single building level^42^, or documented accuracies in building delineation are low especially in these complex areas (e.g.^43–46^). No globally systematized and highly accurate compendium or subset of poverty areas at the single-building scale has ever been released. Our dataset provides manually interpreted/delineated building footprints, covering 321,075 single buildings plus 4,661 building block polygons. It covers 44 areas classified as being poor, i.e. documented by other literature, from major cities across the globe. In 16 of these areas, we provide additional multitemporal data demonstrating the spatial evolution over time.

Complemented by metadata, interpretation guidelines, and uncertainty documentation, this contribution closes a critical gap in openly available geodata of difficult-to-access and difficult-to-map areas. By making these data accessible, we support the global systematization and comparative analysis of settlement forms representing poverty, support the training and validation of automated methods, and provide a robust empirical basis for advancing settlement geography and remote sensing research.

## Methods

### Data sources

**Fig. 1:**
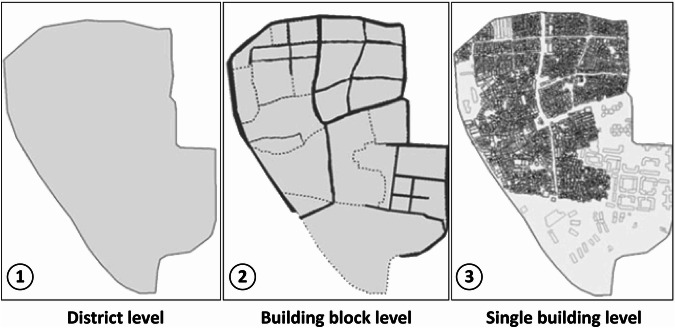
Spatial scope: 1. District level (entire area), 2. Building block level and 3. Single building level, based on “*The physical face of slums*”^18^.

The geo-dataset^41^ contains building footprints, building blocks and entire district areas (cf. Figure 1). It was derived from visual interpretation of very high resolution (VHR) optical satellite imagery, primarily by QuickBird, GeoEye and WorldView, provided by ©Google Earth and ©European Space Imaging, with ground sampling distances (GSD) of approximately 0.5 meters. The basemaps were temporarily used, remain in our local environment and are not part the published data.

Our dataset contains ©OpenStreetMap building footprints and ©ESRI Basemap building footprints that were all revised, respectively changed by the human interpreter. The data provides sufficient spatial detail to identify individual rooftops, even in dense urban fabrics. In our approach, we found a minimum mapping unit of 4 m^2^ appropriate, taking into account the geometric resolution for urban building delineation which is controversially discussed and changes in research history due to evolving technologies^47–51^. Imagery was selected to minimize cloud cover and maximize interpretability. Acquisition dates span the period from ~2002 to 2017, depending on availability for the selected sites.

We delineate the spatial scale on three levels:

- *District level* (entire area or ‘Area Of Interest (AOI)): This is a unit representing a poverty area within an urban area and can be based on administrative boundaries. Some areas are as large as 2 km^2^, others are small covering only 0.2 km^2^. Due to comparability and resource limits, we did not always capture the whole district unit and chose an AOI, that represents all given morphological structures. Because of internal physical variation, only general morphological features can be studied at this level.
- *Building block level*: The street networks usually form blocks subdividing urban areas that tend to show more uniform physical features than larger districts. In poverty areas, however, dense construction and missing or ambiguously identifiable pathways render streets alone insufficient for the identification of blocks. Therefore, to delineate blocks, structural change-overs, e.g. from dense to low dense settlements or to open spaces are further considered and manually added, if applicable.
- *Single building level*: Object-specific parameters can be characterized (e.g. shape, size, height) to analyze the morphology at the finest scale of the three levels. The lack of reliable building-level data for poverty areas remains a major limitation in remote sensing and geographical research, resulting in a widespread reliance on aggregated representations of the built environment. The dataset presented in this publication contributes to reducing this gap.

A total of 44 poverty areas were selected covering 35 cities across Africa, Asia, North and South America, and Europe. The choice of areas was guided by six criteria: 1. *morphological and topographical (terrain) diversity*, capturing settlement types ranging from dense inner-city slums to peri-urban informal neighborhoods and transitional forms; 2. *global representativeness*, ensuring coverage of major cities in different world regions; 3. *data availability*, requiring access to VHR imagery suitable for building-level mapping; 4. including cases with varying *degrees of formality*, planning, and socio-economic context; 5. *Documented in literature as* urban *poverty area* (thus, an intensive literature research was part of the applied method, cf.^4^); 6. an accumulation of at least *1,000 buildings* understood as settlement.

For 16 of all the poverty areas, additional building footprints and building blocks were generated for a second distinct time step [t0; t1]. Acquisition years typically ranged from 2002 to 2017, depending on image availability. This design enables the assessment of structural changes over approximately one decade. Unlike many large-scale approaches, no seasonal harmonization was performed. Thus, vegetation and illumination conditions may differ between t0 and t1. This variability can – unlike algorithmically – be equalized by an experienced interpreter. The VHR imagery allows reliable identification of building footprints. A complete overview of all areas and multitemporal sites is provided in the Data Records section: Study Sites and data volume.

### Manual visual image interpretation (MVII)

**Fig. 2:**
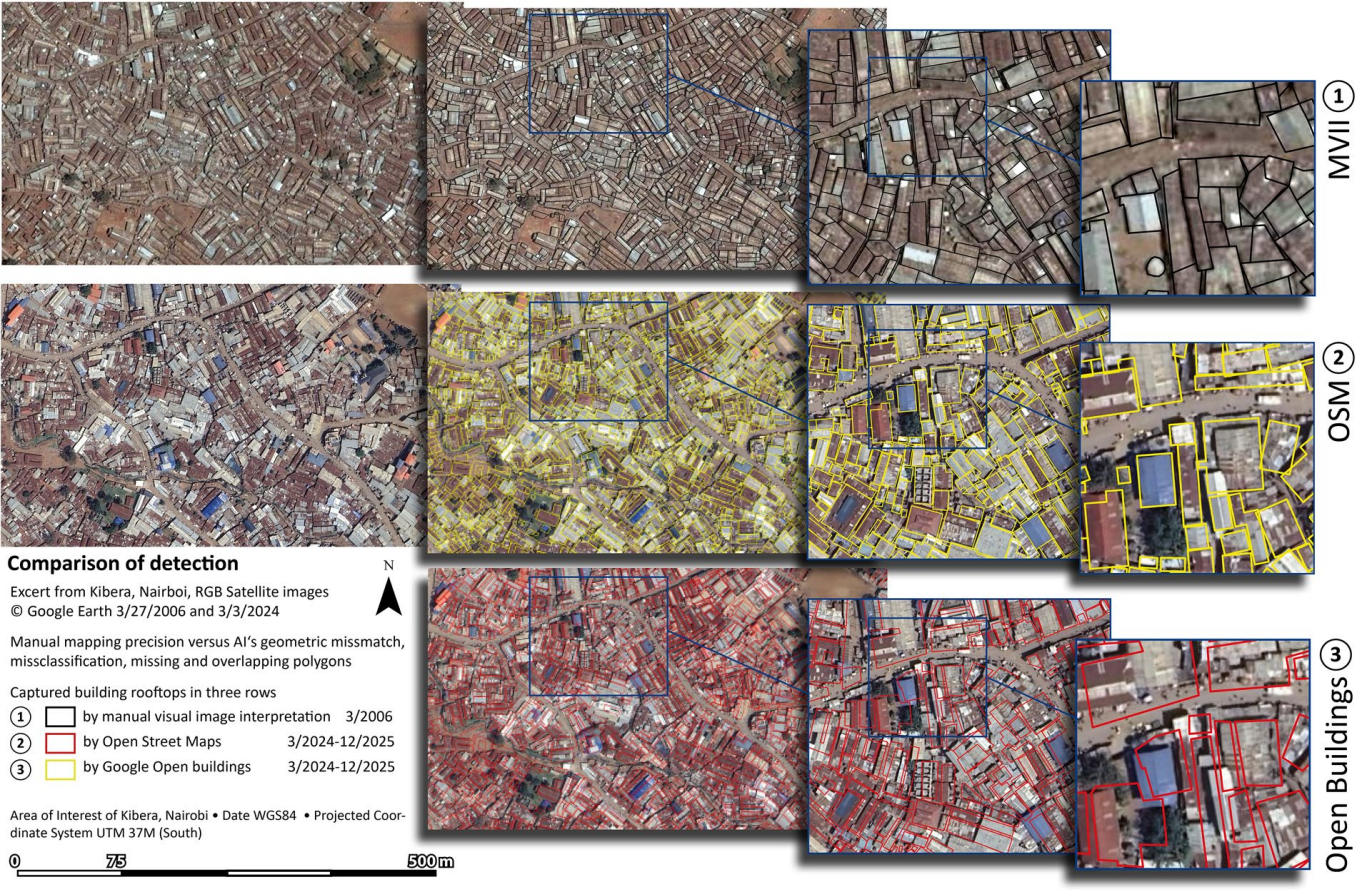
Excerpt from Kibera, Nairobi’s largest slum: Captured building rooftops showing a uniform, textured pattern. Manually mapped by MVII from the year 2006 in comparison to ©Open Street Maps^77^ and ©Google Open building datasets^46^ from, the year 2025 with obvious inaccuracies.

The underlying approach represents one of the oldest and still most robust approaches for extracting information from remotely sensed imagery. The method has been applied since the early 1970s, when very high-resolution aerial and medium to low resolution satellite data first became available^52^. This approach is based on systematic image interpretation elements such as tone, color, texture, shape, size, pattern, shadow, and spatial context. These elements form the cognitive framework of human perception in image analysis^53,54^ and enable interpreters to cognitively reconstruct the built environment even in complex urban fabrics, where spectral similarity or occlusion typically challenges automated methods (Fig. 2). Interpreters benefit from the human brain ability to transform image data from their visual appearance into geodata proxies using the wealth of their experience of the real world’s building structure. Thus, the process is inherently cognitive and relies on interpreter’s subjective experience to disambiguate visual cues in dense, irregular morphologies^53^^p. 131^. Due to more modern machine and artificial intelligence (deep learning) approaches in remote sensing, MVII is nowadays only applied in 10% of poverty area research^3^.

### Mapping protocol and criteria of classification and delineation

**Fig. 3:**
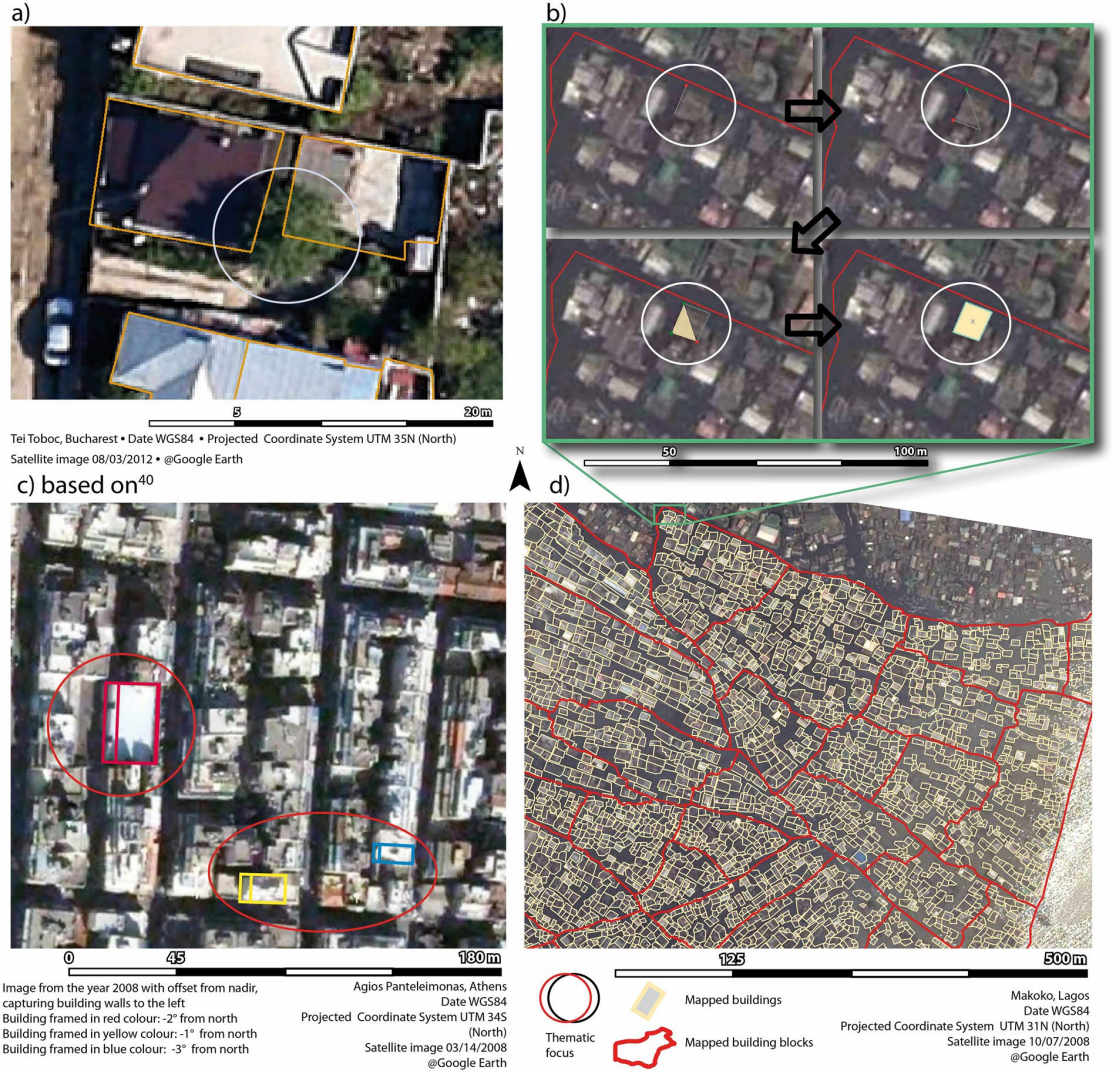
(**a**) Due to the ‘amodal completion’, cognition allows to set vertices even in hidden allocations in Bucharest; (**b**) Mapping process placing vertices step by step to gain a rooftop polygon in Lagos; (**c**) Examples of nadir offset at building balconies in Athens; (**d**) Building block containing LoD-1 building footprints that allow inter- and intra-urban analyses in Lagos.
- In case of partial occlusion (e.g., by shadows or vegetation), the most probable geometry was reconstructed by ‘amodal completion’, drawing on visual interpretation principles (contrast, line continuation, texture, context) (cf. Figure 3a).
- Potentially ambiguous cases (e.g., narrow gaps between roofs, blurred edges) were resolved by analyzing local texture and spatial patterns according to the mapping protocol.
- We mapped polygons without overlaps or gaps; curved rooflines were approximated by short linear segments. A general rule was to map as tightly to the edge as possible, provided the edge was unambiguous, in order to guarantee constant building size digitization.
- In dense roofscapes, where individual buildings could not be separated with confidence, we generalized polygons to represent building clusters, provided the rule was applied consistently within the AOI.
- We were aware of off-nadir effects, i.e. rooftops might be distorted, facades are visual. Balconies have not been mapped as building (cf. Figure 3c). Despite consistent inclusion or exclusion decisions across the AOI occasionally produced geometric distortions cannot be precluded.

In some areas, existing OpenStreetMap and ESRI building footprints were used as a starting point, and some areas were initially mapped by different interpreters (cf. Supplementary Table 1). However, most areas were mapped by a single image interpreter, who also carried out the final harmonization of the entire dataset, including areas initially mapped by other interpreters. Harmonization followed the standardized MVII protocol^4,22^ and included systematic verification of polygon vertices and polygon placement to ensure geometric consistency with the reference imagery. Where necessary, polygons were extensively revised to ensure consistent application of the mapping criteria throughout the dataset. The standardized mapping protocol ensures reproducibility across study sites and facilitates reproducibility across interpreters. Accordingly, building footprints were delineated and mapped following the steps below:

1. We firstly mapped rooftops as two-dimensional Level of Detail (LoD)-0 polygons with a minimum mapping unit of ~4 m^2^ that match the aforementioned sufficient geometric resolution (cf. section Methods: Data sources).

- We mainly performed the mapping at a working scale of ~1:1,000 based on VHR satellite imagery as aforementioned (cf. section Methods: Data sources). Zooming was used flexibly for better detection results during mapping.
- Vertices were placed directly on clearly identifiable rooftop edges (eaves, ridges, corner points), with polygon segments following the visible rooftop boundary (cf. Figure 3b). Depending on the building morphology, we place at least 4 or even more vertices to reach the exact LoD-1.2 as defined by^55^. For simplicity, we remain naming it LoD-1.

2. We secondly derived the ‘Building height’ from ancillary sources e.g., shadow analysis, oblique view, spatial structures and patterns, field surveys, or street-view photography and an estimation approach based on Kraff, 2011^39^. With it, we gained the third dimension LoD-1.
3. In our study in^4^, we aggregated the single building data of the first two steps (cf. the hierarchy in fig. 1.3), to block scales (1.2) and to district scales (1.1), enabling the calculation of spatial variables (*density, orientation, heterogeneity, size, height*) consistently across scales. To do so, an additional mapping of ‘*building blocks’* was adopted to gain another spatial level. In this case vertices were drawn along streets and pathways to aim at a block containing the afore mapped single buildings (cf. Figure 3d) for intra-urban analyzes on district level. This spatial unit allows to set buildings in relation to the block and receive follow-up spatial variable calculations as e.g., *building densities* per block. Further, blocks can be set in relation to each other. In this vein, intra-urban analyses are possible based on single building features. Thus, we delineated *building blocks* and mapped them by the following steps:

- Vertices were placed directly along clearly identifiable pathways, streets or similar bordering building structures.
- We cut main streets or waterbodies and drew manual boundaries at obvious structural transitions (e.g. open spaces) to define blocks with more homogeneous physical traits.
- For further intra-urban analyses and statistical comparability, we mapped the blocks in relatively similar sizes. If block sizes outraged in size due to the above-mentioned procedure, we drew lines along building’s edges to delineate and facilitate block size comparability.

4. For the *multitemporal* approach in our study^22^, we applied the same mapping protocol to identical AOI boundaries at two different time steps. Acquisition years typically ranged from 2002–2010 (t0) to 2011–2020 (t2). Seasonal variation in vegetation was disregarded, reflecting a defined constraint of the dataset because vegetation can influence building delineation.

Uncertainty is quantified using inter-rater reliability as a proxy for mapping consistency in the absence of an independent ground truth (cf. section Technical Validation and uncertainty assessment of Manual Visual Image Interpretation). For those study sites previously investigated by^40^, the corresponding inter-rater reliability values are provided as quantitative proxy measures of mapping uncertainty (cf. Supplementary Table 1).

The reproducibility discussed in this study refers to the manually delineated individual building footprints, which constitute the core component of the published dataset. Building blocks represent a secondary aggregation of these footprints and, although based on expert interpretation, are delineated according to a predefined rule-based mapping scheme described by^4^.

## Data Records

### Format

We provide the complete geo-dataset^41^ as Shapefile (.shp) with the typical accompanying projection and attribute files (.dbf,.prj,.sbn,.sbx,.shp.xml). Although the Shapefile format has widely been replaced by GeoPackage, it remains one of the most universally recognized and interoperable standards for vector geodata across GIS platforms, including older systems. It can easily be transferred to Geopackage. Other than raster, these vector files allow highest quality for further processing because they are independent of scale. We systematically offer three data-sets (cf. Table 1): (1) LoD-1 single building vector polygons; (2) building block vector polygons; (3) entire poverty area respectively the AOI.

**Table 1:** Attribute tables of single buildings, building blocks and the entire/Area of Interest.

Explanation of attribute fields	LoD-1 “housing_[*poverty area name]*.shp”	Building block “block_[*poverty area name]*.shp”	Entire poverty area “AOI_[*poverty area name]*.shp”
FID	Standard attribute table entry equals each building polygon ID	Standard attribute table entry, equals key for building block	Standard attribute table entry, no equation
Shape	Standard attribute table entry	Standard attribute table entry	Standard attribute table entry
ID	Key for building block in which the single building polygon lies entirely inside	Key for building block	Only one ID entry for the entire area
Area	Calculated m^2^ for each building polygon	Calculated m^2^ for the building block	Entire Area Of Interest, calculated m^2^, based on the specific location
	UTM WGS84 projection, based on the specific location e.g. areas in Cario: 36 R (North)		
Height	Estimated building storeys: 2: building with 1-2 storeys 5: building with 3–5 storeys 8: building with 6–8 storeys 11: building with 9–11 storeys 12: building with 12 and more storeys	—	—
Formality (only for Mumbai areas)	0 = informal 1 = formal	0 = informal 1 = formal	Covers both, formal and informal area

Metada/attributes include ‘ID’; ‘Shape’; ‘Area’ (m^2^) of buildings, blocks and the AOI; ‘Height’ of buildings (storeys); and, for Mumbai, we offer an additional formal dataset, thus there is a ‘Formality’ status entry. Formality refers to the documented formal or informal status of a settlement according to the literature, particularly with respect to land tenure, legal recognition, and planning status.

All data can be downloaded here: https://download.geoservice.dlr.de/BUILDING_FOOTPRINTS/files/

For further information, cf. section „Data Availability Statement”.

### Exemplified application of building footprint polygons: Criteria

In the mentioned studies^4,18,22^, we used the created data to analyze the morphological structures of poverty areas. We selected variables that are commonly applied in urban morphological research (e.g.^8,56,57^). We additionally created variables specifically to obtain spatiotemporal and intra-urban details. Thus, we calculated the variables on two spatial levels a) from the mapped footprints and b) from the building blocks containing the building footprints:

- ‘Number of buildings’
- ‘Building size’ (footprint area, in sqm)
- ‘Building orientation’ (long-axis direction, in degrees)
- ‘Building density’ (footprint area of all buildings per defined block area in percent)
- ‘Built-up heterogeneity’ (density differences between building blocks, as arbitrary unit).

The provided building footprints allow users to derive all building-based morphological metrics used in our previous (multi-temporal) morphology classification studies (e.g., building density, mean building size, or the standard deviation of building size aggregated at the building block level). Details of these derivations are provided in the Code Availability section, where we exemplify some mathematical calculations how to compute the spatial variables. More details can be found in the method sections of our publications^4,18,22,40^.

### Study sites and data volume

Mapping is highly time-consuming, and achieving comparability in terms of size, morphological representativeness, boundary uncertainty, and minimum building count is challenging. For these reasons, we selected ‘Areas Of Interest’. Whenever possible, we mapped the ‘Entire Area’ (EA), each at the district level (cf. Figure 1).

Our basic population of geodata contains AOIs and EAs representing a specific point in time and may be subject to boundary changes over time. For simplicity, we consistently use the term AOI and labelled the files accordingly. AOI’s either have a specific local name, otherwise we choose the district name or used attached street names. We delineate an AOI based on typical morphological features representing poverty: narrow pathways, dense and complex building patterns, a coherent morphology and open spaces, but we exclude formal areas. In some cases, we slightly extended AOI boundaries, e.g. in case of unclear historical administrative boundaries. Thus, some AOIs are even larger than the administrative boundary (for instance in Eldarb el-Ahmar). We list the complete overview of all study area information in Supplementary Table 1. In total 321,075 building polygons were mapped. We additionally captured 4,661 building blocks and added a merged dataset for the district level (cf. spatial levels Fig. 1).

Working in a consistent methodological manner, the overall pattern changes only slightly. So, the built-environment required only rarely different intra-urban block delineations. Thus, the number of building blocks differs only in few areas over time (cf. Supplementary Table 1). References documenting the poverty areas are listed in Supplementary Table 1. Further contextual background information can be found in appendices of^4,22^.

**Fig. 4:**
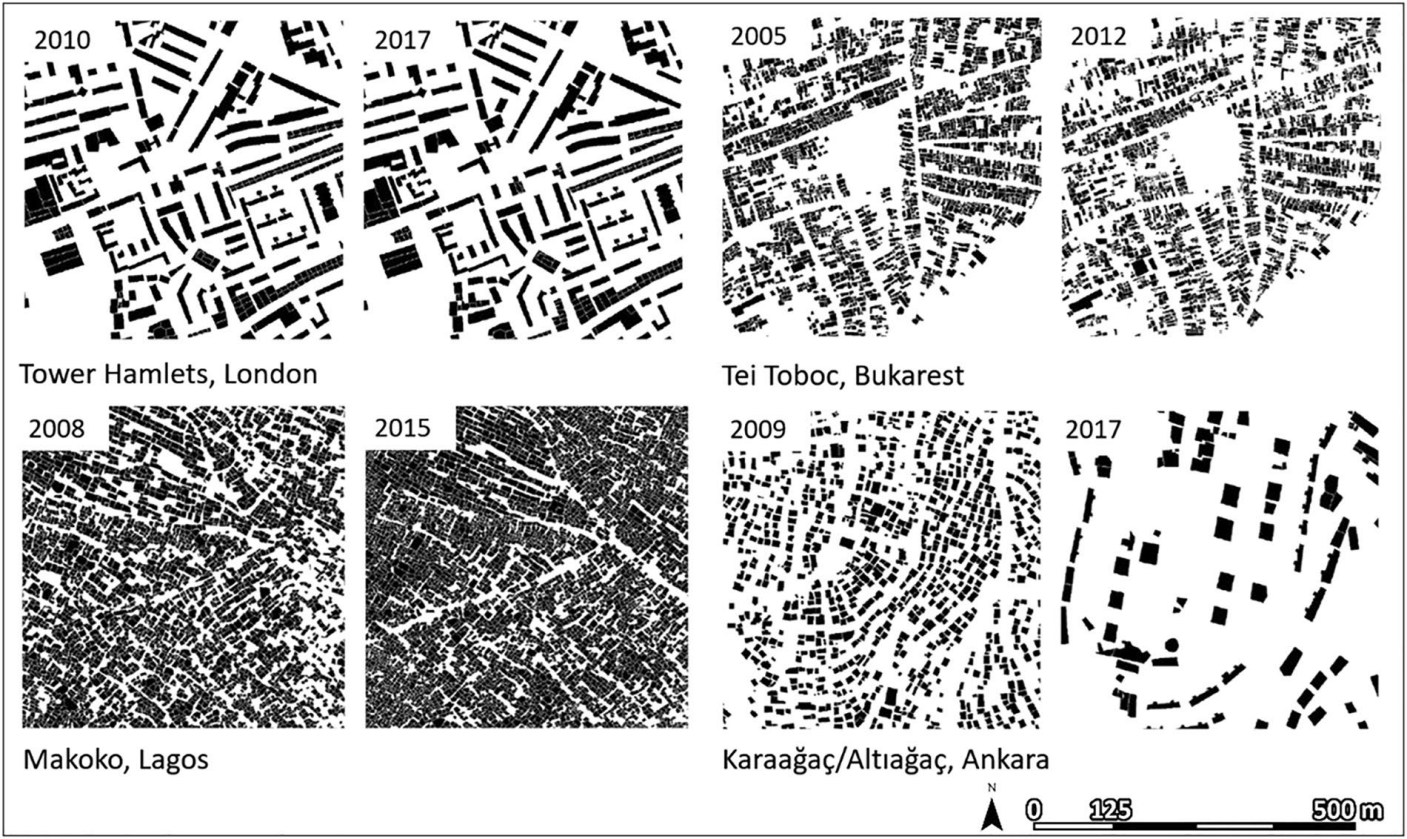
Excerpt of study sites: Ground figure plans, adapted from “*The Morphology of the Arrival City*”^4^ and from “The dynamics of poor urban areas”^22^) derived from multitemporal optical satellite imagery by ©Google Earth.

We used single building analyses to understand morphological appearances of poverty. In our publications^4,18,22,40^, the basic population of the study sites demonstrated e.g. a wide variation in building density, size, and orientation (cf. Figure 4), with forms ranging from very dense, organically developed structures to more loosely organized, geometrically planned layouts. High densities tend to occur with small buildings. The visual diversity shows that one cannot assume a single, uniform morphology for areas inhabited by the urban poor. High dynamics can be found especially in the Global South (e.g. in Makoko, Lagos, Nigeria, cf. Figure 4) but also in cases of governmental reordination programs (e.g. Karaağaç/Altıağaç, Ankara, Turkey, cf. Figure 4).

This final dataset^41^ is the result of a controlled MVII process combined with a rule-based harmonization procedure, ensuring internal consistency across interpreters, while uncertainty is characterized through inter-rater reliability as a proxy for mapping consistency. The following section provides a technical validation of this consistency.

## Technical Validation and uncertainty assessment of Manual Visual Image Interpretation

To evaluate the reproducibility of MVII, systematic interpreter tests were conducted in^40^. Multiple interpreters mapped identical areas, and outputs were compared using overlap measures such as intersection-over-union and Fleiss’ Kappa. Results revealed a high consistency in one interpreter’s mapping behavior on several areas. However, several interpreters map differently, so there is also a significant inter-rater variability.

### Rationale for MVII and ensuring data quality

MVII retains a central position in Earth observation, particularly in contexts where automated classification techniques encounter limitations. Even though automated methods for building detection and extraction have improved, they still remain prone to errors in morphologically complex poverty areas with spectral similarity, shadowing, and interwoven roof structures^27–29^. Figure 2 demonstrates that automatically detected publicly available datasets have hardly been refined, even with image qualities superior to those from nearly 20 years ago. In contrast, human interpreters are capable of synthesizing a broad range of visual cues – including shape, texture, shadows, spatial relationships, and contextual patterns. This allows interpreters to benefit from the brain’s ability to transform image data and their wealth of experience of building structures in the real world.

**Fig. 5:**
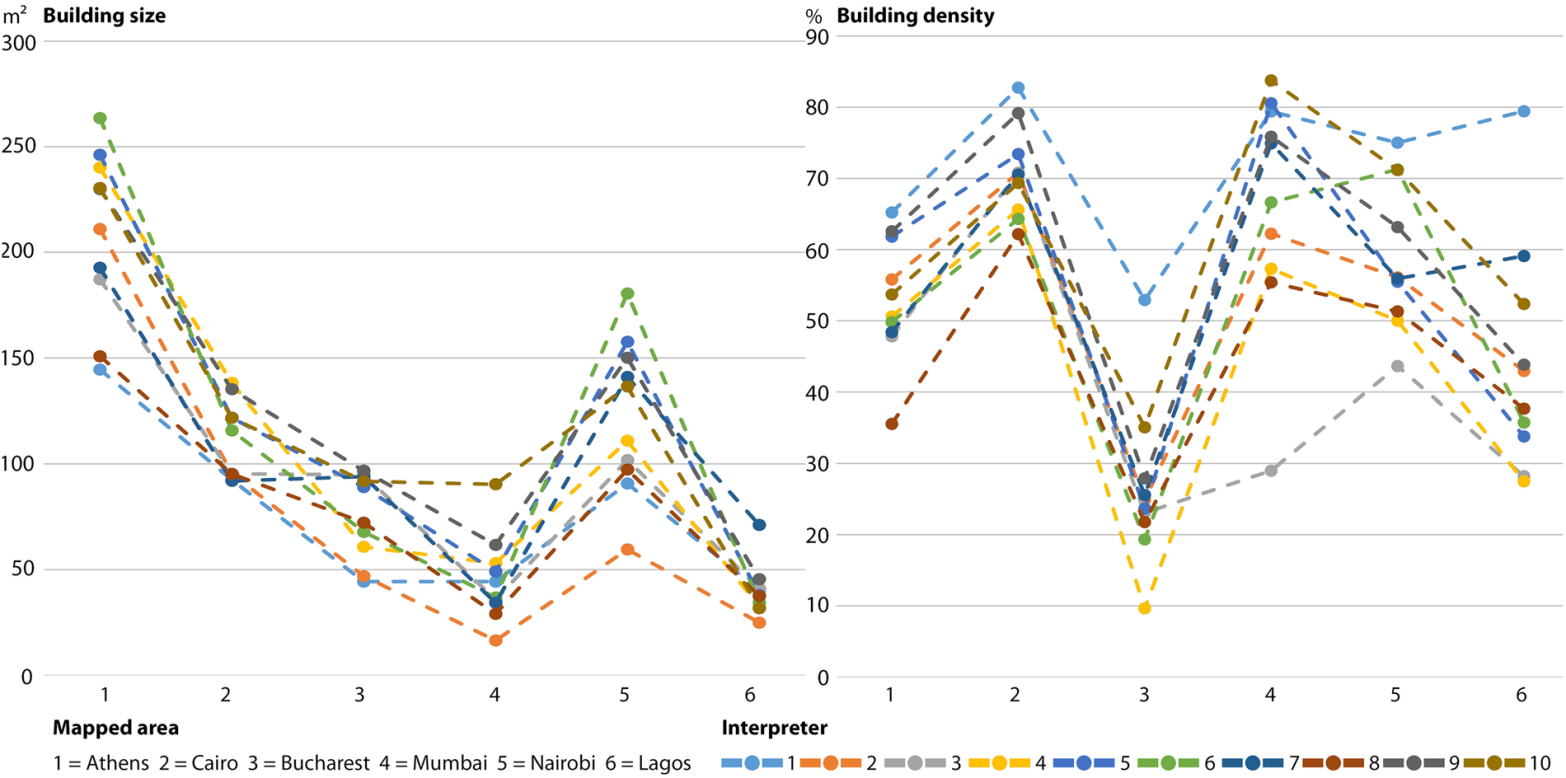
Consistent mapping of building sizes and building densities. Adapted from “*Uncertainties of human perception in visual image interpretation in complex urban environments*”^40^.

In complex built environments, such as informal settlements or irregularly structured historic quarters, this interpretive flexibility enables the detection of subtle or fragmented features that automated systems may overlook: At highly detailed spatial scales (LoD-1), where individual buildings are delineated, MVII can yield semantically meaningful representations of the urban fabric. One interpreter constantly places vertices precisely and in the same way along visible edges, ensuring robust building-level geometries. It serves as a reliable empirical basis for settlement morphology analyses. So, MVII benefits from strong internal consistency at the individual level. While results may vary between interpreters, a single person typically applies their interpretation strategy systematically and uniformly across tasks (cf.^40^). This internal stability reduces within-dataset variability and supports reproducible outcomes, which is particularly advantageous when one interpreter maps an entire study area (cf. Figure 5).

Another important strength of this method lies in the adaptability of interpreters: they can dynamically modify zoom levels, compensate for visual distortions, and draw upon contextual reasoning—for example, distinguishing shadows from actual structures or recognizing recurring roof patterns as indicative of residential clusters. Further, visual perception offers central *cognitive* advantages: so-called “tolerance” and “amodal completion” help to identify buildings on the strength of habit and experience, e.g., in case of incomplete treetop covering (cf. Figure 3a^58^). MVII therefore continues to provide the most accurate reference data for single-building mapping in poverty areas (cf. Figure 2). The standardized protocol presented in the section Methods: Mapping protocol and criteria of classification and delineation, ensures globally comparable building footprint data, supporting both urban morphology research and the development of automated classification algorithms. However, a real ‘ground truth’ of the exact building morphology remains unknown in these areas.

### Uncertainties of MVII

Although MVII offers several advantages, it is inherently prone to uncertainty due to its subjective nature. There are hardly studies on how well reality is reproduced as *ground truth* is commonly not registered due to missing official (e.g. cadaster) information. However, empirical studies have documented substantial variation between individual interpreters, particularly when working with imagery of complex urban landscapes. In one of our mentioned studies, we applied an accuracy assessment for three less complex and three more complex environments^40^. To assess interrater reliability, we examined how accurately interpreters placed polygons on building rooftops. We created a grid with 2x2m per cell (4 m^2^ minimum mapping unit) for each area. Each interpreter’s dataset was spatially joined with this grid, and every intersected cell was counted as a match. We then compared all cell matches and calculated agreement using the Fleiss-Kappa index, where 0 indicates poor and 1 almost perfect agreement between interpreters. The results revealed moderate agreement for less complex areas (κ ≈ 0.4–0.6) and only fair agreement for more complex areas (κ ≈ 0.3–0.4) cf. Supplementary Table 1.

In further comparative mapping exercises, coefficients of variation (CV) for parameters such as building counts and footprint sizes frequently ranged between 20% and 30%. These discrepancies persisted even under standardized mapping protocols, demonstrating the strong influence of interpreter-specific characteristics, including prior GIS expertise, experience with urban morphology and specially with complex urban environments, and familiarity with the imagery – as reported by the interpreters. Qualitative evidence highlights the perceptual dimension of this variability: Interpreters often reported being affected by factors such as brightness, contrast, shadow effects, and atmospheric distortions. Homogeneous rooftop textures or vegetation cover frequently increased ambiguity and led to divergent mapping results. Fatigue also emerged as a relevant factor: with mapping durations ranging from less than five to over eleven hours, many interpreters experienced noticeable declines in attention and precision over time. Several reported applying less detail in later tasks or relying more heavily on expectations formed during previous mapping steps.

These findings indicate that, despite its interpretive flexibility and high level of detail, MVII lacks full reproducibility and is susceptible to both perceptual and procedural influences. Consequently, the use of MVII for validation, training data generation, or analytical purposes should be accompanied by explicit uncertainty assessments, standardized procedures, and, where appropriate, multi-annotator approaches to enhance the robustness and reliability of results. For all details, we refer to^40^. Recent work by^59^ supports the characteristics of manual visual image interpretation in informal settlement mapping, highlighting strong inter-annotator agreement in well-defined core areas and increased variability in peripheral regions, thereby reinforcing the need for a standardized and clearly defined mapping protocol.

## Usage Notes and Discussion

Geodata are always subject to uncertainty^60^ and the *ground truth* of poverty related housing constructions is largely unknown. Even though the human cognition reaches its limits^59,61^ and demonstrates significant deviations between interpreters^40^, urban remote sensing studies commonly refer to manually mapped building information because it is considered closest to the ground truth^62–64^. Along with this consensus, we argue that MVII products such as these presented in this publication are of high value when reliable official data is unavailable and especially when serving as labels for machine learning algorithms^31^. We further encourage future studies producing MVII derived datasets to encompass further reliability assessments to better inform the end-users.

Publications of the last years on poverty areas, slums or informal settlements demonstrate a significant research cluster in urban morphology research^65^. The research community aims at a global sum repository^3,14^, which, however, is still absent. This ambitious goal requires globally systemized quantifiable (reference) data^66^, in contrast to studies with qualified descriptive output (e.g.^2,8^). To respond to SDG 11.1.1., a deep understanding of the urban morphology in poverty areas is necessary. These areas commonly lack official statistical and administrative data (e.g., cadaster, census, formality status and so on^4,5^). For global data on building delineation, systematizing human map creation remains a utopia. Considering the scale of the task and the temporal validity, it is clear that only automatic delineation can serve this goal. Earth observation offers the nationwide data capabilities. Large scale or even global automatically generated 3D-building stocks of formal settlements already exits, e.g., at continental scale^67^, or globally by the “Global Human Settlement Layer”^68^, the “Global Urban Footprint”^41^, the “World Settlement Footprint 3D”^69^, Google’s^47^ “OpenBuildings2.5D” or the “Global Building Atlas”^45^. However, the complex informal fabric remains hardly capturable, remains relatively inaccurate (cf. e.g. Figure 2), or is not explicitly stated in these data records. Still, machine learning procedures and artificial intelligence approaches have demonstrated the feasibility to capture single buildings even in highly complex poverty areas, if training data sets are available^70^. Even though scalable and transferable approaches to map deprived areas are still limited^71^, the current literature suggests that the future of large-scale or even global building detection lies in deep learning. Thus, our open data set serves as a basic step towards a global systematization and comparative analysis of poverty settlement forms. Besides the sole morphological perspective, where remote sending reaches its limits, poverty area research demands *in-situ* data. Only a combined perspective of household data linked with remote sensing data allows a full understanding in the context of settlement geography. Only this allows a concrete application for decision makers or planners^72^. Especially high-resolution data as single buildings can serve more valuable information, when linked to micro census household data, rather than aggregated buildings stock data. However, household data contain sensitive information and must be handled with care to prevent stigmatization. Thus, geo-ethical aspects need to be taken into account: personal information must not be traced, as due to security, privacy, personal data as well as personal rights and possession, especially in areas being prone to dwelling^11,73–76^. In our study, we rely on published documented areas and mostly freely accessible image data. None of the building polygons can be traced back to any personal information.

## Supplementary information

Supplementary Material_Table1

## Data Availability

The geo-dataset described in this Data Descriptor is publicly available through the German Aerospace Center (DLR) and is referenced in Ref. 41. The data are released under a Creative Commons Attribution (CC BY) license. Following publication, the dataset will be accessible via the DOI provided in Ref. ^41^. OSM Building footprints were obtained from OpenStreetMap (©OpenStreetMap contributors, https://www.openstreetmap.org), which is a collaboratively maintained, community-generated geospatial database.Building footprints were manually mapped using the ESRI World Imagery basemap as the reference imagery under a valid ArcGIS license. The published vector dataset is an original product of manual visual image interpretation and does not contain or redistribute proprietary ESRI imagery.

## Code availability

No executable code is provided for this study. Our geo-dataset^41^ is based on building footprints. It was generated through a manual, interpretation-based geospatial workflow involving visual digitization of polygon geometries from imagery rather than an automated or script-based processing pipeline. As a result, the data creation process is not implemented in code and cannot be meaningfully reproduced through software execution.

The subsequent analytical use of the dataset is fully specified through the mathematical formulations listed below. These define all derived variables in a software-independent manner and are presented as exemplary applications of these formulas.

LoD-1 polygon calculate field of square meters (m^2^), based on UTM WGS84 projection. Example for a GIS Python Code:

import arcpy

in_fc = r“C:\Path\to\your\layer.shp” # or feature class in a geodatabase

field_name = “Area_m2”

# add

arcpy.AddField_management(in_fc, field_name, “DOUBLE”)

# calculate area

arcpy.CalculateField_management(

in_fc,

field_name,

“!shape.area@SQUAREMETERS!”,

“PYTH7ON3”)

We offer examples for the application and calculation of spatial variables for the single building polygons on the building block level:

1. no. of buildings = n

L = spatial level building block $n=\sum \left({polygons}\,{in}\,L{\rm{\_}}1\right)$

2. Average building size (m^2^) = *G*

g = Rooftop surface equals ground floor (m^2^) $G=\frac{{\sum }_{n=1}^{{n}_{{L}_{1}}}{g}_{n}}{{n}_{{L}_{1}}}$

3. Average building height (storeys) = *H*

h = single building height (storeys) ${\rm{H}}=\frac{{\sum }_{n=1}^{{n}_{{L}_{1}}}{h}_{n}}{{n}_{{L}_{1}}}$

4. Average building orientation = *O*

r = building orientation (°) abs. value [−90; 90] $O=\frac{{\sum }_{n=1}^{{n}_{{L}_{1}}}{I}_{n}^{r}}{{N}_{{L}_{1}}}$

main (long) edge orientation of the polygon relative to geographic north

*r*^*N*^ building orientation (°) of nearest neighbor (nn)

Δ^*r*^ difference [0; 90] to nearest neighbor

→ Resulting in *I*^*r*^ as normalized index for a pair of buildings

(building and its nn) to a morphological structure

].

5. Ø building density (%) = *D*

A = area of the block (m^2^) $D=\frac{{\sum }_{n=1}^{{n}_{{L}_{1}}}{g}_{n}}{{A}_{{L}_{1}}}$

6. Temporal dynamics, can be used for any parameter = T ${\rm{T}}=\frac{{t}_{1}-{t}_{0}}{{t}_{0}}\times 100$

For more details we rely on^4,18^ (especially intra-urban heterogeneity)^22^ for the spatiotemporal analyses.
