# Supplementary material for: A manually interpreted geo-dataset of building footprints in complex urban poverty areas across the globe
Source: Sci Data. 2026 Aug 3;13:1120. doi: 10.1038/s41597-026-07945-2 (PMC13434708; doi:10.1038/s41597-026-07945-2)
Supplement: Supplementary file 1 [file 41597_2026_7945_MOESM1_ESM.docx]

**Supplementary Material**

**Supplementary Table 1:** Basic population of study sites across the world, no. of polygons per area, data record, projection and documented poverty proof, based on^10^.

| **Poverty area / Study site**  alphabetical ordered by continents, countries, cities and area | **UTM-Zone projection** | **Recording/Basemap date** [dd.mm.yyyy]  16x two time steps (t_0_ +t_1_), all other data rely on one (later) time step | **District level**  [km²] contains the entire poverty area (EA) or a representative part (AOI) (km²)  T_0_ + eventually t_1_ | **Manual mapping by**  **image interpreter** | **Mapped no. of buildings** (polygons)  T_0_ and eventually t_1_ | **Mapped no. of building blocks**  (polygons)  t_0_ and eventually t_1_ | **Example literature verification** that proofs area as poor  [cf. literature reference in main article] | **Inter-rater reliability values** demonstrated for selected poverty areas, based on^40^:  Fleiss-Kappa [0;1] for geometric polygon-match;  and agreement-range [%], based on four spatial variables. |
| --- | --- | --- | --- | --- | --- | --- | --- | --- |
| **Africa** | | | | | | | |  |
| 01. Eldarb el-Ahmar, Cairo, Egypt, Africa | 36R (N) | 06.12.2011 | 0.92, AOI | DLR internal mapping.  Revised and refined by N. Kraff | 7,334 | 72 | 78, 79, 80 | - |
| 02. Ezbet el-Haggana, Cairo, Egypt | 36R (N) | 06.12.2011 | 1.26, EA |  | 9,228 | 81 | 2, 81 | - |
| 03. Imbaba, Cairo, Egypt | 36R (N) | 01.07.2004 t_0_ | 0.46, AOI | N. Kraff | 2,052 | 61 | 2, 82 | [0.42]  and [81-91%] |
|  |  | 12.06.2011 t_1_ | 0.46, AOI | DLR internal mapping.  Revised & refined by N. Kraff | 3,341 | 61 |  |  |
| 04. Manshiet Nasser, Cairo, Egypt | 36R (N) | 2014 | 0.81, AOI |  | 8,943 | 69 | 83 | - |
| 05. Kibera, Nairobi, Kenya | 37M (S) | 27.03.2006, t_0_ | 0.51, AOI | N. Kraff | 4,254 | 33 | 2, 84 | [0.43]  and [64-95%] |
|  |  | 24.05.2014, t_1_ | 0.51, AOI |  | 7,529 | 33 |  |  |
| 06. Makoko, Lagos, Nigeria | 31N (N) | 07.10.2008, t_0_ | 0.53, EA |  | 6,462 | 42 | 85, 86 | [0.36]  and [63-82%] |
|  |  | 17.01.2015, t_1_ | 0.50, EA |  | 9,651 | 42 |  |  |
| 07. Lafoole Refugee Camps, Afgooye, Somalia | 38N (N) | 05.01.2011 | 0.24, EA |  | 5,142 | 79 | 87, 88, 89 | - |
| 08. Griffiths Mxenge (T1&T2)/Silver Town (T2), Khayelitsha, Cape Town,  South Africa | 34H (S) | 09.07.2005, t_0_ | 0.32, EA |  | 2,403 | 57 | 90, 91 | - |
|  |  | 15.02.2012, t_1_ | 0.32, EA |  | 8,704 | 122 |  |  |
| 09. Mandela Park, Khayelitsha, Cape Town, South Africa | 34H (S) | 05.04.2012 | 0.23, AOI |  | 1,237 | 33 |  |  |
| 10. Victoria Merge, Khayelitsha, Cape Town, South Africa | 34H (S) | 28.06.2013 | 0.13, AOI |  | 2,268 | 15 |  |  |
| **America** | | | | | | | |  |
| 11. Turano, Rio de Janeiro, Brazil | 23K (N) | 25.09.2002 t_0_ | 0.34 | N. Kraff | 3,297 | 53 | 92, 93 | - |
|  |  | 25.06.2009 t_1_ | 0.33, EA |  | 5,010 | 53 |  |  |
| 12. Paraisópolis, São Paulo, Brazil | 32K (S) | 15.12.2008 | 0.81, EA |  | 26,934 | 45 | 94, 95 | - |
| 13. Lo Prado, Santiago de Chile, Chile | 19H (S) | 2016 | 0.22, AOI | DLR internal mapping.  Revised & refined by N. Kraff | 1,854 | 22 | 96, 97 | - |
| 14. Magdalena Contreras, Mexico City, Mexico | 14Q (N) | 10.04.2015 | 0.75, AOI | N. Kraff | 9,848 | 103 | 98, 99 | - |
| 15. Comas/Independencia, Lima, Peru | 18L (S) | 02.03.2009 t_0_ | 0.35, AOI |  | 3,497 | 62 | 100, 101 | - |
|  |  | 01.03.2016 t_1_ | 0.42, AOI | DLR internal mapping.  Revised & refined by N. Kraff | 3,681 | 74 |  |  |
| 16. West Adams, Los Angeles, USA | 11S (N) | 2016 | 0.55, AOI | N. Kraff | 3,546 | 59 | 16, 102 | - |
| 17. Bronx, New York City, USA | 18T (N) | 21.02.2009 t_0_ | 2.16, AOI | N. Kraff | 3,424 | 182 | 103, 104 | - |
|  |  | 26.06.2016 t_1_ | 2.17, AOI | DLR internal mapping, based on  ESRI* World Imagery.  Revised & refined by N. Kraff | 3,208 | 182 |  |  |
| 18. Hunting Park/Juniata Park, Philadelphia, USA | 18T (N) | 12.04.2010 t_0_ | 1.75, AOI | N. Kraff | 7,231 | 171 | 105, 106 | - |
|  |  | 21.09.2017 t_1_ | 1.75, AOI | DLR internal mapping, based on  ESRI* World Imagery.  Revised & refined by N. Kraff | 7,211 | 171 |  |  |
| 19. Petare, Caracas, Venezuela (Barrio 5,12, 19) | 19P (N) | 22.03.2014 | 0.42, AOI | N. Kraff | 6,247 | 21 | 107, 108 | - |
| **Asia** | | | | | | | |  |
| 20. Islambag, Dhaka, Bangladesh | 46Q (N) | 2010 | 0.12, EA | Mapping by Ines Standfuß.  Revised & refined by N. Kraff | 1,441 | 54 | 109, 110 | - |
| 21. Kadamtali/Chunkutia, Dhaka, Bangladesh | 46Q (N) | 2010 | 0.58, AOI |  | 3,025 | 147 | 111, 112 | - |
| 22. Dashilanr/Chunshu/Liuxue Road Community, Beijing, China | 50S (N) | 30.09.2016 | 1.56, AOI | N. Kraff | 16,131 | 114 | 113, 114 | - |
| 23. Baishizhou, Shenzhen, China | 49Q (N) | 20.02.2008 t_0_ | 0.39, EA |  | 2,002 | 18 | 115, 116 | - |
|  |  | 23.01.2015 t_1_ | 0.38, EA |  | 1,944 | 18 |  |  |
| 24. Bharat Nagar (Chembur), Mumbai, India | 43Q (N) | 25.01.2010 | 0.17  (formal + informal), EA |  | 4,354 (formal + informal) | 11  (formal + informal) | 17, 117 | - |
| 25. Dharavi, Mumbai, India | 43Q (N) | 25.01.2010 | 1.99 (formal + informal), EA |  | 42,455  formal + informal) | 65 (formal + informal) | 118, 119 | - |
| 26. Santosh Nagar (Goregaon), Mumbai, India | 43Q (N) | 12.10.2003 t_0_ | 0.23, EA |  | 3,371 | 16 | 117, 120 | [0.34]  and [52-92%] |
|  |  | 25.01.2010 t_1_ | 0.41 (formal + informal), EA |  | 4,581  (formal + informal) | 26  (formal + informal) |  |  |
| 27. Eslamshahr, Tehran, Iran | 39S (N) | 09.11.2016 | 1.48, AOI |  | 10,938 | 189 | 121, 122 | - |
| 28. Refugee Camp, Al-Zaatari, Jordan | 37S (N) | 2013 | 1,14, AOI | DLR internal mapping.  Revised & refined by N. Kraff | 8,435 | 185 | 87, 123 | - |
| 29. Khoroo 9, Ulaanbaatar, Mongolia | 48T (N) | 13.04.2007 t_0_ | 0.67, EA | N. Kraff | 4,233 | 87 | 124, 125 | - |
|  |  | 20.11.2014 t_1_ | 0.70, EA |  | 4,960 | 90 |  |  |
| 30. North Cemetery, Manila, The Philippines | 51P (N) | 22.03.2016 | 0.18, AOI |  | 15,563 | 69 | 126, 127 | - |
| 31. Tondo/San Nicolas, Manila, The Philippines | 51P (N) | 07.08.2015 | 0.20, EA |  | 7,065 | 36 | 128, 129 | - |
| 32. Karaağaç/Altıağaç, Ankara, Turkey | 36S (N) | 02.07.2009 t_0_ | 0.81, EA |  | 2,198 | 100 | 130, 131 | - |
|  |  | 21.09.2017 t_1_ | 0.85, EA | DLR internal mapping.  Revised & refined by N. Kraff | 639 | 84 |  |  |
| 33. Gülsuyu-Gülensu, Istanbul, Turkey | 35T (N) | 27.05.2012 | 1.36, EA | N. Kraff | 7,166 | 140 | 132, 133 | - |
| 34. Kadifekale, Izmir, Turkey | 35S (N) | 01.02.2013 | 0.36, EA | DLR internal mapping.  Revised & refined by N. Kraff | 3,055 | 72 | 134, 135 | - |
| **Europe** | | | | | | | |  |
| 35. Molenbeek, Brussels, Belgium | 31U (N) | 18.08.2016 | 1.26, AOI | DLR internal mapping.  Revised & refined by N. Kraff | 5,771 | 113 | 136, 137 | - |
| 36. Nadezhda, Sliven, Bulgaria | 35T (N) | 22.04.2014 | 0.19, EA |  | 1,910 | 68 | 138, 139 | - |
| 37. Tower Hamlets, London, England/UK | 30U (N) | 20.09.2008 t_0_ | 2.18, AOI | N. Kraff | 2,449 | 101 | 16, 140 | - |
|  |  | 04.06.2015 t_1_ | 2.18, AOI | OSM** building footprints,  revised by N. Kraff | 2,508 | 101 |  |  |
| 38. Belleville/Combat/La Banane, Paris, France | 31U (N) | 06.04.2014 | 1.49, AOI |  | 3,029 | 82 | 141, 142 | - |
| 39. Les Pyramides, Évry, France | 31U (N) | 12.06.2005 t_0_ | 2.40, AOI | N. Kraff | 1,315 | 71 | 16, 143 | - |
|  |  | 31.12.2011 t_1_ | 2.40, AOI | OSM** building footprints,  revised by N. Kraff | 1,270 | 71 |  |  |
| 40. Kreuzberg, Berlin, Germany | 33U (N) | 07.06.2016 | 3.42, AOI | N. Kraff | 4,275 | 132 | 16, 144 | - |
| 41. Agios Panteleimonas, Athens, Greece | 34S (N) | 14.03.2008 | 0.71, EA |  | 2,806 | 158 | 145, 146 | [0.59]  and [74-89%] |
|  |  | 13.04.2016 | 0.68, EA |  | 3,017 | 153 |  |  |
| 42. Cova da Moura, Lisbon, Portugal | 29S (N) | 06.08.2015 | 0.14, EA | DLR internal mapping.  Revised & refined by N. Kraff | 936 | 21 | 147, 148 | - |
| 43. Tei Toboc, Bucharest, Romania | 35T (N) | 25.06.2005 t_0_ | 0.28, EA | N. Kraff | 2,981 | 31 | 149, 150, 151 | [0.49]  and [27-87%] |
|  |  | 03.08.2012 t_1_ | 0.28, EA |  | 3,805 | 31 |  |  |
| 44. Cañada Real Galiana, Madrid, Spain | 30T (N) | 27.08.2011 | 0.39, EA |  | 2,804 | 74 | 152, 153 | - |

* Esri. World Imagery basemap^153^. Esri, Redlands, CA, USA. Available from Esri ArcGIS Online.

**Building footprints were obtained from OpenStreetMap^55^ (©OpenStreetMap contributors, https://www.openstreetmap.org), which is a collaboratively maintained, community-generated geospatial database.

List of references according to column “Example literature verification”

78. Abouelmagd, Shaimaa. (2013). Geographical Information System an approach for the Development of Slums and Deteriorating areas in Cairo, Egypt. ISBN: 978-90-365-0747-9
79. UCLG, Committee on Social, Inclusion, Participatory Democracy and Human Rights. Inclusive Cities Observatory (2014): Cairo, Egypt: The al-Darb al-Ahmar Housing Rehabilitation Programme
80. Taha, A. M., Bahgat, H. A., & Ghafaar, A. M. A. (2022). An Approach to Prioritize Urban Conservation Heritage Areas According to Social Indicators. Civil Engineering and Architecture, 10(5), 10-13189.
81. Boules, D. E., Sherif, L. A., & Khalil, M. H. (2020). Residential typologies as a tool to trace the urban transformation of Ezbet El-Haggana. J. Eng. Appl. Sci, 67, 1285-1304.
82. Harris, R., & Wahba, M. (2002). The urban geography of low‐income housing: Cairo (1947–96) exemplifies a model. International Journal of Urban and Regional Research, 26(1), 58-79.
83. El Araby, M. (2002): Urban growth and environmental degradation – The case of Cairo, Egypt. Cities, Vol. 19, No. 6, p. 389–400, 2002
84. Veljanovski, T., Kanjir, U., Pehani, P., Oštir, K., & Kovačič, P. (2012). Object-based image analysis of VHR satellite imagery for population estimation in informal settlement Kibera-Nairobi, Kenya. In Remote Sensing-Applications. InTech.
85. Gambo, Y. L., Idowu, O. B. & Anyakora, I. M. (2012): Impact of Poor Housing Condition on the Economy of the Urban Poor: Makoko, Lagos State in View. Journal of Emerging Trends in Economics and Management Sciences (JETEMS) 3(4): pp. 302-307
86. Babalobi, B. (2013): Water, sanitation and hygiene practices among primary-school children in Lagos: a case study of the Makoko slum community. Volume 38/7 Pp. 921-929, Taylor & Francis
87. Schöpfer, E., Pelizari, P. Aravena Pelizari & Spröhnle, K. (2015). Temporäre Siedlungen: Wenn aus Flüchtlingslagern Städte werden. 71-81. 10.1007/978-3-662-44841-0_9.
88. Spröhnle, K., Tiede, D., Schöpfer, E., Füreder, P., Svanberg, A., & Rost, T. (2014). Earth observation-based dwelling detection approaches in a highly complex refugee camp environment—A comparative study. Remote Sensing, 6(10), 9277-9297.
89. OCHA - UN Office for the Coordination of Humanitarian Affairs (2009): Map of Somalia: Afgooye Corridor and Kax-Shiiqaal - Identification of New Settlements, January 2008 - January 2009, 25 August 2009, http://www.refworld.org/docid/4aa79b21d.html
90. University of Cape Town, Southern Africa Labour and Development Research Unit (2000): South Africa - Khayelitsha Mitchell's Plain Survey 2000. KMP 2000 Report. Online source: http://www.datafirst.uct.ac.za/dataportal/index.php/catalog/4
91. Khayelitsha Community Trust (2011): Annual Report. https://www.google.com/url?sa=t&source=web&rct=j&opi=89978449&url=https://resource.capetown.gov.za/documentcentre/Documents/City%2520research%2520reports%2520and%2520review/KCT_Annual_Report_2010_11.pdf&ved=2ahUKEwjR9cOX5_GQAxXd_7sIHZrjFCYQFnoECBcQAQ&usg=AOvVaw1mzcb5rB1S-OU65F8AKjdM
92. Portes, A.. (1979). Housing Policy, Urban Poverty, and the State: The Favelas of Rio de Janeiro, 1972-1976. Latin American Research Review, 14(2), 3–24. http://www.jstor.org/stable/2502876
93. Cavallieri, F., Vial, A. (2012): Favelas na cidade do Rio de Janeiro: o quadro populacional com base no Censo 2010. COLEÇÃO ESTUDOS CARIOCAS. IPP/Prefeitura da Cidade do. Rio de Janeiro.
94. Sanches, C. (2013): A Tool for bridging the formal-informal divide in São Paulo. In: Coelho L, Melgaco L, Muguia R (2013): Mundus Urbano: (Re)thinking urban development. Frank & Timme GmbH. pp. 117f.
95. Taubenböck H & Wurm M (2013): Die Frucht des Paradieses – Reflektionen über den Besuch einer Favela in Sao Paulo. Standort. Vol. 37, issue 4. pp. 270-275.
96. Government of Chile (2006): Casen 2006 Région Metropolitana. Encuesta de caraterización socioeconómica nacional. Online source: https://web.archive.org/web/20070824153157/http://www.mideplan.cl/final/bajar.php?path=casen2006regional&id=CASEN2006-Metropolitana.pdf
97. Cummings, J., DiPasquale D. (1997): The Spatial Implications of Housing Policy in Chile. – In: City Research 1997.
98. Aguilar, A. G. and Guerrero, F. L. (2013): Poverty in Peripheral Informal Settlements in Mexico City: The Case of Magdalena Contreras, Federal District. Tijdschrift voor economische en sociale geografie, 104: 359–378. doi: 10.1111/tesg.12012
99. Connolly, P. (2003): Urban Slums Reports: The case of Mexico City, Mexico - In: UN-Habitat: UNDERSTANDING SLUMS: Case Studies for the Global Report on Human Settlements 2003
100. Riofrío, G. (2003): Urban Slums Reports: The case of Lima, Peru - In: UN-Habitat: UNDERSTANDING SLUMS: Case Studies for the Global Report on Human Settlements 2003
101. Plöger, J. (2012). Gated barriadas: Responses to urban insecurity in marginal settlements in Lima, Peru. Singapore Journal of Tropical Geography, 33(2), 212-225.
102. City of Los Angeles Department of City Planning (2008): Profiles of General Demographic Characteristics. 2000 Census of Population and Housing
103. Bloch, M., Ericson, M., & Giratikanon, T. (2014): Mapping poverty in America: Data from the Census Bureau show where the poor live. New York Times. Online Source: http://www.nytimes.com/newsgraphics/2014/01/05/poverty-map/ (16.09.2017).
104. U.S. Census Bureau (2016): Bronx County (Bronx Borough), New York. Population estimates, July 1, 2016. Online source: https://www.census.gov/quickfacts/fact/table/bronxcountybronxboroughnewyork/EDU685215
105. Jargowsky, P. A. (1997). Poverty and place: Ghettos, barrios, and the American city. Russell Sage Foundation.
106. City of Philadelphia (2016): Assessment of Fair Housing. Online source: http://www.pha.phila.gov/media/170834/assessment_of_fair_housing_2016-web.pdf
107. Ramos, C., Vicentini, A., Ortega, D. (2012): Challenges and Opportunities of Waste Collection in Caracas: Sucre Municipality Case Study. Columbia University Academic Commons, http://dx.doi.org/10.7916/D8H131Q0.
108. Marques, B. P., & Carvalho, R. (2010): Policies to improve the lives of Slum Dwellers–from international agreements to local contexts. The Brazilian case-study. In Atas do 16. º Congresso da APDR (pp. 554-580). APDR-Associação Portuguesa para o Desenvolvimento Regional.
109. Etzold, B. (2011): Street Food Governance in Dhaka (Bangladesh): The Appropriation of Street Vending Spaces in Dhaka and the Informal Politics of Exploitation. RC21 Conference 2011 “The struggle to belong - Dealing with diversity in 21st century urban settings”,  Amsterdam, July 7-9 2011.
110. Hackenbroch, K. (2013). The spatiality of livelihoods-negotiations of access to public space in Dhaka, Bangladesh. Steiner.
111. Raihan, M. M. H., Islam, M. N., Rouf, A., Begum, A., Rahman, M., Murad, M., & Das, S. (2014). Health Care Situation of Migrant Slum Women: Evidence from Sylhet City of Bangladesh. Bangladesh e-Journal of Sociology, 11(1).
112. Standfuß, I. (2012): The Physical Growth of Informal Settlements in Dhaka, Bangladesh - A spatio-temporal case analysis based on high-resolution satellite data from 2006 and 2010. Unpublished diploma thesis. TU Dortmund University.
113. Zhao, P. 2016: The Disappearing Historical Hutongs: Key Issues in Preserving the Locality in Old Beijing. – In: Wang, F., & Prominski, M. (2016). Urbanization and Locality. Springer.
114. Rock, M. Y. (2012). Splintering Beijing: Socio-spatial fragmentation, commodification and gentrification in the hutong neighborhoods of'old'Beijing. The Pennsylvania State University.
115. Qu, L., & Van Dorst, M. J. (2014). Perceived Control and Liveability: Environment and behaviour interaction in two urban villages of Shenzhen.
116. Zhang, L., Zhao, S. X. B. and Tian, J. P. (2003). Self-help in housing and chengzhongcun in China's urbanization. International Journal of Urban and Regional Research, 27(4), 912-37.
117. Government of Maharashtra (2007): Maharashtra Disaster Management Plan. Greater Mumbai Disaster Management Action Plan. Risk Assessment and Response Plan. Vol.1. Mumbai. http://www.mcgm.gov.in/irj/portalapps/com.mcgm.aDisasterMgmt/docs/Volume %201 %20 %28Final %29.pdf
118. Desai, V. (1988). Dharavi, the largest slum in Asia: Development of low-income urban housing in India. Habitat International, 12(2), 67-74.
119. Sharma, K. (2000). Rediscovering Dharavi: stories from Asia's largest slum. Penguin Books India.
120. Risbud, N. (2003): Urban Slums Reports: The case of Mumbai - In: UN-Habitat: UNDERSTANDING SLUMS: Case Studies for the Global Report on Human Settlements 2003
121. Hosseinioon, S. (2014). Informality on the edge: an insight into the formalisation of informal settlements in Tehran conurbation, Iran. National Library of Australia Cataloguing-in-Publication entry, 22.
122. Samiei, A., & Sayafzadeh, A. (2016): Analysis of the Worn-Out Tissues Characteristics and Providing of Intervention Pattern, Case Study: Eslamshahr City, Tehran. Current Urban Studies, 4(03), 267.
123. Ledwith, A. (2014). Zaatari: The Instant City. Affordable Housing Institute.
124. Batayev, Z. (2014): Verstädterung in der Mongolei: unter besonderer Berücksichtigung der mongolischen Hauptstadt seit der Jahrtausendwende.
125. UNICEF (2003): The living conditions of the children in peri-urban areas of Ulaanbaatar. Summary Report.
126. Aoki, H. (2008): Globalization and the Street Homeless in Metro Manila – in: PHILIPPINE STUDIES 56, no. 1 (2008): 69–76
127. Au, M. (2016). Housing the Dead: A lense through local densification patterns. Room One Thousand, 1(4).
128. Philippine Sociological Society (1968): Squatting and Slum Dwelling in Metropolitan Manila Special Committee Report. Philippine Sociological Review Vol. 16, No. 1/2 (JANUARY-APRIL, 1968), pp. 92-105 Published by: Philippine Sociological Society Online source: http://www.jstor.org/stable/41853556
129. Ragragio, J. M. (2003): Urban Slums Reports: The case of Metro Manila, Philippines - In: UN-Habitat: UNDERSTANDING SLUMS: Case Studies for the Global Report on Human Settlements 2003
130. Duyar-Kienast, U. (2005): The formation of gecekondu settlements in Turkey: the case of Ankara (Vol. 7). LIT Verlag Münster.
131. İdel, C. (2018). Hegemonic politics of homeownership and formation of neolibera subjectivieis; former gecekondu residents in Gülveren - Ankara (Master Thesis at the Graduate School of Social Sciences of the Middle East Technical University).
132. Özdemır, E. (2013): Contesting Neoliberal Urbanization: Contemporary Urban Movements in İstanbul, the Case of Gülsuyu–Gülensu Neighborhoods. Unpublished PhD Thesis, METU, Ankara.
133. Adanali, Y.,Balanli, I. (2009): Interview with Erdogan Yildiz. In - Istanbul Living in Voluntary and Involuntary Exclusion (2009). Pp. 13-14
134. ÇETĠN, Ġ. (2016). An examination of informal settlements (gecekondus) in the concept of sociology of space: case of Izmir. *Humanitas*, *4*(7), 115-125.
135. Demirli, M. E., Ultav, Z. T., & Demirtaş-Milz, N. (2015). A socio-spatial analysis of urban transformation at a neighborhood scale: The case of the relocation of Kadifekale inhabitants to TOKİ Uzundere in İzmir. Cities, 48, 140-159.
136. Buffel, T., Phillipson, C., & Scharf, T. (2013): Experiences of neighbourhood exclusion and inclusion among older people living in deprived inner-city areas in Belgium and England. Ageing & Society, 33(1), 89-109.
137. deDecker, P., & Kesteloot, C. (2002): Migration, Housing and Homelessneess in Belgium.
138. Ladányi, J., & Szelényi, I. (2001): The social construction of Roma ethnicity in Bulgaria, Romania and Hungary during market transition. Review of Sociology, 7(2), 79-89.
139. Zhelyazkova, A., Daskalova, N., & Nazarska, G. (2003). Racial and ethnic minorities, immigration and the role of trade unions in combating discrimination and xenophobia, in encouraging participation and in securing social inclusion and citizenship.
140. Kearns, K. C. (1979). INTRAURBAN SQUATTING IN LONDON∗. Annals of the Association of American Geographers, 69(4), 589-598.
141. Aguilera, T. (2013): Configurations of squats in Paris and the Ile-de-France Region. Squatting in Europe: Radical Spaces, Urban Struggles, 209.
142. Simon, P. (2000). The mosaic pattern: cohabitation between ethnic groups in Belleville, Paris. In Minorities in European cities: the dynamics of social integration and social exclusion at the neighbourhood level (pp. 100-115). London: Palgrave Macmillan UK.
143. INSEE - Institut national de la statistique et des études économiques (2017): POP T0 - Population par grandes tranches d'âges. Online Source: http://www.insee.fr/fr/statistiques/2011101?geo=COM-91228#chiffre-cle-1
144. Stehle, M. (2006): Narrating the Ghetto, Narrating Europe: From Berlin, Kreuzberg to the Banlieues of Paris – IN: Westminster Papers in Communication and Culture.
145. Kandylis, G., & Kavoulakos, K. I. (2011): Framing urban inequalities: Racist mobilization against immigrants in Athens. Επιθεώρηση Κοινωνικών Ερευνών, 136(136).
146. Kalantzopoulou M., Koutrolikou P., Polihroniadi K., 2011, “The dominant discourse about the city-centre of Athens”, Announcement in the discussion organized by the Encounter Athens research group, “Which ‘crisis’ in the centre of Athens? Critical discourse and claims for an equitable city”, NTUA, 16/5/2011.
147. Horta, A. P. B. (2006): Places of resistance: Power, spatial discourses and migrant grassroots organizing in the periphery of Lisbon. City, 10(3), 269-285.
148. Ascensão, E. (2013): Following engineers and architects through slums: the technoscience of slum intervention in the Portuguese-speaking landscape. Análise Social, 48(206).
149. Iacoboaea C (2009a): Slums in Romania. In: Theoretical and Empirical Researches in Urban Management Number 1(10)/2009.
150. Iacoboaea C (2009b): Slums in Romania – Case Study – Residential Quarter located on the outskirts of Bucharest City. Metalurgia
151. Teșliuc, E., Grigoraș, V., & Stănculescu, M. S. (2016). The atlas of rural marginalized areas and of local human development in Romania.
152. Gonick, S. (2015): Interrogating Madrid's “Slum of Shame”: Urban Expansion, Race, and Place‐Based Activisms in the Cañada Real Galiana. Antipode, 47(5), 1224-1242.
153. ESRI. World Imagery basemap. Esri, Redlands, CA, USA. Available at: <https://www.arcgis.com/home/item.html?id=10df2279f9684e4a9f6a7f08febac2a9> (accessed 06/2016 (New York City) and 09/2017 (Philadelphia)).
